# Pre-Exposure Prophylaxis Use Among Cisgender and Transgender Adult Entertainment Workers in Brazil

**DOI:** 10.3390/ijerph22081164

**Published:** 2025-07-23

**Authors:** Policardo Gonçalves da Silva, Lariane Angel Cepas, Isadora Silva de Carvalho, Álvaro Francisco Lopes de Sousa, Guilherme Reis de Santana Santos, Caíque Jordan Nunes Ribeiro, Ana Paula Morais Fernandes

**Affiliations:** 1 Department of General and Specialized Nursing, Ribeirão Preto College of Nursing, University of São Paulo, Ribeirão Preto 14040-903, SP, Brazil; goncalvespolicardo@live.com.ar (P.G.d.S.); larianeangelcepas@usp.br (L.A.C.); isadoracarvalho@usp.br (I.S.d.C.); anapaula@eerp.usp.br (A.P.M.F.); 2 Institute of Teaching and Research, Hospital Sírio-Libanês, São Paulo 01308-050, SP, Brazil; 3 Public Health Research Centre, Comprehensive Health Research Center (CHRC), NOVA National School of Public Health, Universidade Nova de Lisboa, 1099-085 Lisbon, Portugal; 4 Graduate Program in Applied Health Sciences, Federal University of Sergipe, Lagarto 49406-584, SE, Brazil; guilheermereeis@gmail.com; 5 Graduate Program in Nursing, Federal University of Sergipe, São Cristóvão 49107-230, SE, Brazil; caiquejordan@academico.ufs.br

**Keywords:** pre-exposure prophylaxis, sex workers, Brazilian unified health system, sexual health, HIV

## Abstract

Adult entertainment work may be associated with increased vulnerability to sexually transmitted infections, particularly HIV. In Brazil, pre-exposure prophylaxis (PrEP) for HIV infection has been available through the Brazilian Unified Health System (SUS) since November 2017, representing a significant advancement in public sexual health policy. The objective of this study was to understand the individual and social determinants that promote PrEP use among adult entertainment workers. This was a cross-sectional, analytical, and quantitative study. A multivariate modeling approach was employed to identify factors independently associated with PrEP use. The study included 254 adult entertainment workers using oral PrEP through the SUS, predominantly young adults (141; 55.5%), SUS users (248; 97.6%), single (213; 83.9%), non-white (142; 55.9%), cisgender (148; 58.3%), and heterosexual (152; 59.8%). Factors independently associated with greater PrEP use included having adult entertainment as the main source of income (aPR: 2.69; 95% CI: 1.86–3.95), prior use of PEP (aPR: 2.49; 95% CI: 1.63–3.81), undergoing any type of health treatment (aPR: 1.56; 95% CI: 1.15–2.12), and having a history of STIs (aPR: 1.51; 95% CI: 1.10–2.08). Conclusion: PrEP use in this population was strongly influenced by structural and contextual factors, indicating that the availability of the technology alone does not ensure its effectiveness.

## 1. Introduction

The human immunodeficiency virus (HIV) remains a global public health issue, with more than 39 million people living with the virus in 2023 [1]. In Brazil, 89,898 new cases of HIV infection were reported between 2022 and 2023, highlighting the urgent need to expand access to public policies for HIV prevention and treatment through the Brazilian Unified Health System (SUS) [2,3]. The country adopts a model based on the principles of universal coverage, ensuring free access to HIV diagnosis, treatment, and prevention, including strategies such as condom distribution, expanded testing for sexually transmitted infections (STIs), post-exposure prophylaxis (PEP), and pre-exposure prophylaxis (PrEP) for HIV [4,5,6].

When used consistently, PrEP can reduce the risk of HIV infection by more than 99%, as demonstrated in studies such as iPrEx, which recorded this level of protection among users who fully adhered to the daily regimen of tenofovir disoproxil fumarate combined with emtricitabine (TDF/FTC) [6]. This efficacy is recognized in the Clinical Protocol and Therapeutic Guidelines for Oral PrEP for HIV Infection, published by the Brazilian Ministry of Health in 2025 [5].

Currently, the SUS provides two PrEP formulations free of charge: TDF/FTC, commercially known as Truvada or available in generic versions, and, since 2023, tenofovir alafenamide combined with emtricitabine (TAF/FTC), the latter recommended preferably for individuals at higher risk of renal or bone adverse effects. Although PrEP is ideally offered at no cost in the public system, it is also available in the private sector, where it entails variable costs that can pose a barrier, especially for populations experiencing social vulnerability or for those seeking confidentiality. However, the existence of PrEP in the SUS does not, in itself, ensure widespread use. Structural barriers, social stigma, socioeconomic inequalities, and logistical challenges, particularly in non-metropolitan areas, are recognized as factors that limit the reach of prophylaxis, especially among more vulnerable groups [1,5].

Furthermore, the use of PrEP requires a certain commitment to regular medical follow-up, which includes renal function assessment, periodic HIV testing, and screening for other STIs [5]. This is partly reflected in data on PrEP use in the country. According to the PrEP Panel of the Ministry of Health, in 2024, 165,473 people obtained the medication at least once from SUS-authorized services. However, only 110,733 remained actively on PrEP as of December, demonstrating significant gaps between availability, adherence, and retention in prophylactic treatment [7].

All of this context makes the discussion of PrEP availability in the Brazilian setting complex and extends beyond merely describing the medications offered. It requires considering the social, economic, and structural contexts that shape real access to and adherence to prophylaxis. This becomes even more critical when considering marginalized populations and barriers they may face in using this strategy.

A group that exemplifies how such barriers intertwine to hinder access to HIV prevention is that of adult entertainment workers (AEWs). Beyond the specific characteristics of key populations, occupational context can also contribute to increased vulnerability to HIV infection—for example, among AEWs, especially those who engage in sexual activity in exchange for money [8]. Although sex work is not criminalized in Brazil, social stigma and marginalization place these professionals at high risk. This is due not only to frequent exposure to multiple sexual partners but also to structural conditions that impede access to health services, such as violence, discrimination, institutional barriers, and economic instability [9,10].

Adult entertainment workers are individuals who work professionally in the sex industry, encompassing a wide range of occupations involving the production, performance, or provision of erotic or sexual content or services. This group includes, but is not limited to, pornographic film actors, erotic content models (including camgirls and camboys), strip club dancers, escorts (sex workers), dominatrices/dominators, and other related professions. These professionals may work either in person or in digital environments, as occurs on adult content platforms that offer on-demand services [11,12].

Social vulnerability is a fundamental determinant for individuals entering this type of work. In Brazil, members of marginalized groups—especially lesbian, gay, bisexual, travesti, transgender, queer, intersex, and asexual (LGBTQIA+) individuals—face significant structural barriers, including educational inequality, discrimination in the formal job market, and social exclusion [13]. The absence of family support and social networks can further exacerbate this vulnerability, leading to financial difficulties and greater dependence on informal or precarious employment. In this context, digital platforms such as OnlyFans, Privacy, and JustForFans have emerged as alternative sources of income for many, offering a pathway to financial autonomy. However, participation in these platforms may involve transitions between digital adult entertainment and in-person sex work, which can increase exposure to HIV and other STIs, particularly in the absence of effective prevention measures [14].

Although they do not constitute a homogeneous group, many AEWs face social and structural vulnerabilities that may elevate their risk of STIs including HIV. Factors such as stigma, discrimination, precarious working conditions, and barriers to accessing health services can hinder effective engagement in preventive strategies such as PrEP [15,16]. Despite PrEP being firmly established as an important public health policy in Brazil, its use and continuity among AEWs are still not fully understood, revealing gaps in knowledge about the individual, social, and structural factors influencing adherence. Even with significant progress in PrEP availability through the SUS, challenges persist, such as lack of knowledge about prophylaxis, logistical barriers to access, cultural factors, and stigma within health services [17,18]. In light of this, the present study seeks to fill this knowledge gap by investigating the factors associated with PrEP use among AEWs.

## 2. Materials and Methods

### 2.1. Study Design

This is a cross-sectional analytical study [19], as data on exposures (sociodemographic, occupational, and behavioral characteristics) and the outcome (PrEP use) were collected simultaneously at a single point in time, without longitudinal follow-up of the participants.

### 2.2. Study Setting

The study was conducted in the city of São Paulo, Brazil, between March and August 2022. Data collection was carried out at a non-governmental organization (NGO) called *Instituto Cultural Barong*, headquartered in São Paulo, Brazil. The NGO is recognized for its significant work in health promotion and the prevention of STIs, HIV/AIDS, and viral hepatitis, offering services such as testing, counseling, and psychological and social support.

### 2.3. Population, Sample, and Eligibility Criteria

The study population consisted of AEWs who sought services at an NGO in São Paulo, Brazil. Participant recruitment employed both convenience sampling and snowball sampling [20], a technique in which new participants are recruited through referrals from those already participating in the study.

Inclusion criteria for participants were being an adult entertainment professional (self-reported), being 18 years of age or older, and being registered with PrEP reference services in Brazil and/or affiliated organizations. Individuals who did not meet these criteria or who declined to participate were excluded from the study.

### 2.4. Data Collection Procedures

Initially, the research team contacted the NGO to present the study objectives, the data collection instruments to be used, and the public health relevance of the project for vulnerable populations such as AEWs. Following a favorable response, data collection began with support from the NGO’s multidisciplinary team, which directed users to a private area before or after their consultations, where they could complete the questionnaire if they consented.

Participants were approached and invited to voluntarily participate in the study after receiving a full explanation of the study’s objectives, potential risks and benefits, and the informed consent process. Data collection was carried out through the administration of a printed questionnaire by the research team, with an estimated duration of 15 min. Additionally, sexual health awareness activities were conducted in open public spaces with high pedestrian traffic in São Paulo, such as Avenida Paulista and Largo do Arouche. No other type of incentive, such as financial compensation, was offered to research participants.

### 2.5. Variables and Data Collection Instrument

The questionnaire used in this study was developed by the research team, based on questions previously employed by the Medication Logistics Control System (SICLOM) of the Brazilian Ministry of Health, the official instrument for monitoring PrEP in Brazil. For validation purposes, the instrument underwent face and content review by five researchers who are specialists in the field of HIV/AIDS [20].

### 2.6. Independent Measures

The independent variables for this study were as follows:

- Sociodemographic: age (in years), gender identity (cisgender, transgender), sexual orientation (heterosexual, homosexual, bisexual, other), type of sexual-affective partnership (steady, casual), self-reported skin color (white, non-white), education (years of schooling), monthly income (categorized), housing situation (owned, rented, provided, homeless), and receipt of government assistance.
- Occupational: exclusive or concurrent engagement in other professional activities, and exposure to workplace violence.
- Behavioral: alcohol and illicit drug use, condom use during sexual encounters (always/sometimes/never), history of STIs, history of PEP use, frequency of HIV testing, and use of other combined prevention strategies.
- Healthcare access: history of medical visits in the past six months, experiences of discrimination in healthcare settings, and connection with specialized HIV care services.

### 2.7. Outcome Measure

The dependent variable was PrEP use (yes/no), defined as continuous use for at least three months.

### 2.8. Data Analysis

Statistical analyses were conducted using IBM SPSS software, version 27.0 (SPSS Inc., Chicago, IL, USA). An initial exploratory analysis was performed to describe the distribution of predictor variables according to PrEP use. Contingency table data were presented as absolute and percentage frequencies, calculated using row percentages to estimate measures of association. For the calculation of relative frequencies of exposure categories and missing data, the sample size (n = 254) was used as the denominator. Only variables with less than 20% missing data were included in the analysis.

The measure of association used in this study was the prevalence ratio (PR), as the prevalence of the outcome of interest exceeded 10%. Crude PRs and their respective 95% confidence intervals (95%CI) were calculated to assess the strength of association between variables. Pearson’s Chi-square test or Fisher’s Exact test was used to evaluate the bivariate association between PrEP use among AEWs and the study’s independent variables. This step was used to select variables meeting the statistical threshold of *p*-value < 0.20 for inclusion in the multivariate model.

The final step of the analysis consisted of multivariate modeling to identify factors independently associated with PrEP use among AEWs. A generalized linear Poisson model with a log link function was used, suitable for count-type outcomes, provided that the assumption of equidispersion (equality between mean and variance) was met. The fit of the data to the Poisson distribution was verified using the Kolmogorov–Smirnov test (*p*-value > 0.05), as well as by assessing equidispersion.

To calculate adjusted prevalence ratios (aPRs) and their respective 95% confidence intervals, the hybrid method of parameter estimation was employed, using robust variance estimators and Type III analysis to test model effects. The Omnibus test was applied to determine whether the final multivariate model significantly explained the variability of factors associated with PrEP use compared to the null model (intercept only), adopting a significance level of 5% (*p*-value < 0.05).

Model fit quality was assessed using the Akaike Information Criterion (AIC), the Bayesian Information Criterion (BIC), deviance, and the log-likelihood, with lower values indicating a better fit to the data. The absence of multicollinearity among independent variables was confirmed by variance inflation factor (VIF) values below 10 and tolerance values above 0.1, ensuring robustness of the coefficient estimates. Finally, the significance of the aPRs in the final model was verified using the Wald Chi-square test, with variables considered statistically significant at *p* < 0.05.

### 2.9. Ethical Considerations

This study followed ethical guidelines for research involving human participants, as outlined in the Declaration of Helsinki and in Resolutions 466/2012 and 510/2016 of the Brazilian National Health Council. It was approved by the Research Ethics Committee of the Ribeirão Preto School of Nursing (EERP), University of São Paulo (USP), Brazil (CAAE number: 34573020.3.0000.5393). Informed consent was obtained from all participants, and all information was anonymized to ensure privacy and confidentiality of the collected data.

## 3. Results

The study included 254 AEWs, with an overall prevalence of PrEP use of 32.6% (n = 83). The majority of participants were between 18 and 30 years old (55.5%), used the SUS (97.6%), were single (83.9%), identified as Black (55.9%), cisgender (58.3%), and heterosexual (59.8%) (Table 1). It was observed that only 25.0% of transgender individuals were using PrEP, compared to 38.5% among cisgender individuals. Additionally, 63.8% had an educational level of less than 12 years of schooling, and 60.2% did not own their own home. Factors independently associated with higher PrEP use included having adult entertainment as the main source of income (aPR: 2.69; 95%CI: 1.84–3.95), a history of prior PEP use (aPR: 2.49; 95%CI: 1.63–3.81), being under medical treatment (aPR: 1.56; 95%CI: 1.15–2.12), and having a history of STIs (aPR: 1.51; 95%CI: 1.10–2.08).

Table 2 presents the distribution of AEWs according to their lifestyle habits and sexual practices. Most participants reported having practiced social distancing during the pandemic (170; 66.9%), received at least three doses of the COVID-19 vaccine (173; 68.1%), and reported alcohol consumption (176; 69.3%), while 145 (57.1%) did not report illicit drug use. Additionally, 124 (48.8%) reported a history of violence, and 211 (83.1%) did not engage in receptive anal intercourse. A total of 161 participants (63.4%) had no history of STIs, while 173 (68.1%) reported the use of other combined prevention strategies. Furthermore, 170 (66.9%) had no history of PEP use, 190 (74.8%) had up-to-date immunizations, 131 (51.6%) were currently under medical treatment, 188 (74.0%) had visited a doctor in the past six months, 132 (52.0%) had not visited a dentist in the past six months, and 196 (77.2%) reported never having experienced violence or discrimination in healthcare settings.

Of the 27 variables subjected to bivariate analysis, 15 were eligible for the multivariate analysis phase based on the statistical criterion of *p*-value < 0.20. Of these variables, seven were related to sociodemographic characteristics, while the remainder pertained to lifestyle habits and sexual practices. Additionally, 9 of the 15 variables showed a statistically significant association with PrEP use, being linked to a higher crude prevalence of this outcome.

Subsequently, a generalized linear Poisson regression model was constructed to identify factors independently associated with PrEP use (Table 3). The final model included five variables: one sociodemographic characteristic, three related to sexual practices or health history, and one adjustment variable. The omnibus test revealed that the multivariate model explained the occurrence of the outcome significantly better than chance (*p*-value < 0.001).

All variables retained in the final model were associated with a higher prevalence of the outcome after adjustment for age. Having a main occupation in adult entertainment (aPR: 2.69; 95% CI: 1.84–3.95; *p* < 0.001), a history of PEP use (aPR: 2.49; 95% CI: 1.63–3.81; *p* < 0.001), currently being under medical treatment (aPR: 1.56; 95% CI: 1.15–2.12; *p* = 0.005), and a history of STIs (aPR: 1.51; 95% CI: 1.10–2.08; *p* = 0.010) were all significantly associated with an increased likelihood of PrEP use.

## 4. Discussion

This pioneering study in the Brazilian context investigated factors associated with PrEP use among AEWs, revealing a relatively low prevalence of current PrEP use (32.6%) despite the high vulnerability of this population. We found that being cisgender, having adult entertainment as the primary source of income, a history of prior PEP use, being under medical treatment, and having a history of STIs were factors significantly associated with higher PrEP utilization. These findings are consistent with previous studies showing that engagement with healthcare services and prior experiences with HIV prevention strategies increase the likelihood of PrEP use [21,22,23]. However, the fact that only 25% of transgender individuals in the sample were using PrEP highlights persistent disparities and structural barriers faced by this group, in line with reports from other national [24,25,26] and international studies [27,28]. Overall, our study provides unprecedented data on a largely understudied population and reinforces the need for targeted interventions to expand PrEP access and adherence among sex workers and transgender individuals.

The estimated prevalence of PrEP use in our sample of AEWs in the city of São Paulo was relatively low, at 32.6%. Although higher than that observed in other socially vulnerable groups, such as men who have sex with men living in Brazil (18.3%) and transgender, travesti, and transsexual populations in various regions (with data ranging from 0.9% in Canada to around 40% in Spain) [29,30,31], this percentage still indicates a suboptimal level of preventive coverage given the high risk of exposure in this population. On the other hand, when compared to the international literature, our result is higher than that found in a study conducted among sex workers in Tanzania, an East African country, which identified only 8.0% initiation of PrEP use over 12 months, despite 98.0% of these women reporting willingness to use prophylaxis [32]. These data suggest that, although there is interest in using PrEP, factors such as stigma, structural barriers, lack of facilitated access, and fear of discrimination may restrict adherence, even among highly vulnerable populations [33,34]. In the Brazilian context, the percentage observed may reflect both recent efforts to expand PrEP through the SUS and persistent limitations related to information, stigma, and the structure of health services aimed at sex workers [35].

Unexpectedly, transgender identity was not associated with higher PrEP use, despite this population being considered a priority for prophylaxis in the Brazilian context [36]. In the analyzed sample, 75% of transgender individuals were not using PrEP. This result is concerning, as it contradicts national and international guidelines that recognize the high vulnerability of this population to HIV infection [5,31,37]. Such a discrepancy highlights structural limitations in strategies for access, implementation, and effectiveness of public policies targeting key populations.

Low adherence to PrEP among transgender individuals may be explained by a multitude of factors, reflecting complex intersections among various forms of oppression, marginalization, and social exclusion. In this context, it is pertinent to discuss this issue in light of intersectionality theory, proposed by Kimberlé Crenshaw, which posits that social markers such as gender, gender identity, sexuality, race, social class, and involvement in sex work do not operate in isolation but overlap and interact, producing unique experiences of vulnerability and inequality [38,39]. This approach enables a deeper analysis of the barriers faced by this population in accessing HIV prevention policies.

In the case of transgender individuals working in adult entertainment, there is a clear “overlap of vulnerabilities,” marked by stigma not only related to gender identity but also to their professional activity, which is often marginalized and stigmatized in the social imagination, even though it is not criminalized under Brazilian law [39,40,41]. Added to these dimensions are institutional barriers common to other key populations, such as institutional violence, fear of exposure, and a history of traumatic experiences within healthcare services. These factors reveal power dynamics in which trans bodies are systematically monitored, pathologized, or rendered invisible, including within the scope of HIV prevention policies [42,43]. Additionally, structural obstacles such as the lack of specific campaigns, low trans representation on healthcare teams, absence of qualified listening, and scarcity of welcoming and specialized services contribute to this gap in access [42].

Therefore, the non-use of PrEP by this population cannot be attributed solely to a lack of information or difficulty physically accessing the medication. Rather, it is primarily an expression of unequal power relations and the persistence of cisnormative and heteronormative structures within healthcare systems [44]. Even with PrEP available through the SUS, its implementation faces symbolic barriers, such as discrimination, moral surveillance, and the medicalization of trans identities. Moreover, for this population, seeking PrEP may represent the risk of unwanted social exposure, making it a complex and sometimes conflicted decision between self-protection against HIV and preserving their social and physical safety [24].

Among those whose primary source of income comes from adult entertainment, the very nature of the profession can result in greater exposure to risky situations, reflecting an increased perception of vulnerability in the face of higher-risk events, such as multiple sexual partners, inconsistent condom use, and episodes of violence during sexual activities [45]. Furthermore, it is relevant to consider recent transformations in the sex work market driven by the rise of digital platforms for adult content, such as OnlyFans, ManyVids, and others [46,47]. In recent years, there has been significant growth in the production and consumption of so-called “amateur” pornography, in which sex workers, often operating independently of traditional studios, have begun to produce, promote, and monetize their own content [46]. This technological and cultural shift has introduced new dynamics into sex work. The practice of “collabs” (collaborations between content creators), for example, involves multiple people, often strangers to one another, generating broader sexual networks with greater potential exposure to HIV and other STIs. Additionally, the demand for “unique” or “exclusive” content in the digital environment has encouraged the filming of scenes involving public sex, group sex, or more extreme practices (such as bareback, bondage, or other high-risk activities). These trends reflect the logic of the digital market, in which the uniqueness of each performer translates into greater visibility and financial gains.

Added to this scenario is the normalization of condomless sex in online pornography. Studies indicate that, in recent years, there has been a significant increase in the production and consumption of sexually explicit media in which condoms are not used, influencing public perceptions of safe sex [46,48,49]. For many consumers, the absence of condoms is perceived as synonymous with “greater intimacy” or “realism,” which can negatively affect preventive behaviors among both viewers and content creators themselves [48,49]. In the case of AEWs, this aesthetic and market-driven pressure may lead them to choose to film scenes without condoms, increasing their risk of HIV exposure and consequently heightening the importance of PrEP as a biomedical prevention strategy.

Therefore, discussing PrEP use among AEWs today requires moving beyond the traditional perspective of in-person sex work. It is necessary to consider the impact of digital platforms, which have not only expanded the economic reach of these professionals but also introduced new vulnerabilities, redefining sexual practices and audience expectations. This contemporary context reinforces the need for public policies and health strategies that are both culturally sensitive and technologically up-to-date, including health education initiatives that address the specific realities of digital sex work and its particularities in terms of risk and prevention.

The findings of our study, which indicate a higher prevalence of PrEP use among individuals with a prior history of PEP use (aPR: 2.49; 95% CI: 1.63–3.81), those undergoing continuous medical treatment (aPR: 1.56; 95% CI: 1.15–2.12), and those with a history of STIs (aPR: 1.51; 95% CI: 1.10–2.08), suggest the existence of a cluster of factors reflecting prior connections with healthcare services. This pattern suggests that previous experiences with the healthcare system, whether due to risk situations (such as the need for PEP) or chronic or infectious conditions (such as STIs), serve as entry points for knowledge about and eventual adoption of PrEP.

The literature supports this relationship between prior access to or engagement with healthcare services and greater PrEP adherence. Studies indicate that continuity of care, the trust established between users and healthcare professionals, and familiarity with services are important determinants in the decision to initiate or maintain prophylaxis [50,51,52]. For example, a study conducted in the United States with 110 cisgender male sex workers demonstrated that interventions based on intensive counseling and facilitation of healthcare service access tripled the likelihood of initiating PrEP compared to the control group [53]. These findings reinforce that counseling and structured follow-up interventions can positively impact adherence to biomedical prevention strategies, particularly among historically marginalized populations.

However, there is an apparent contradiction in our study: although 97.6% of the sample relies on the SUS, only 33.5% (n = 83) were using PrEP. This gap challenges fundamental principles of the SUS, such as universality, comprehensiveness, and equity, which entail not only physical access to services but also the provision of approachable, longitudinal, and continuous care. In other words, having access to the SUS alone does not guarantee the effective use of preventive technologies like PrEP.

Several studies help explain this phenomenon by indicating that institutional barriers, stigma, and misinformation continue to shape the relationship between sex workers and healthcare services [54,55,56]. In Germany, research involving healthcare professionals revealed a tendency to overestimate the prevalence of diseases, particularly mental health disorders such as post-traumatic stress disorder, anxiety, and depression, among sex workers [57]. Such perceptions contribute to the construction of stigmas that drive these individuals away from services or make them feel judged and dehumanized. Similarly, a study conducted in South Africa found that the lack of a clear distinction between PrEP and antiretroviral drugs used to treat people living with HIV (PLHIV), combined with limited knowledge about PrEP among healthcare professionals, significantly contributed to the stigmatization of users, creating barriers both to access and adherence [58].

### Recommendations for Public Policy Formulation

Our findings point to the urgent need to rethink public policies aimed at HIV prevention among AEWs. First and foremost, it is essential to recognize that the very concept of AEWs is still evolving and has been undergoing profound transformations in contemporary times. The rise of digital platforms has expanded the traditional boundaries of sex work, creating a new context in which body, intimacy, and market coexist in territories that were once separate.

This transformation can be understood through the lens of Zygmunt Bauman’s theory of liquid modernity, according to which social relationships, identities, and even bodies are becoming increasingly fluid, unstable, and subject to reconfiguration [59,60]. What was once understood as “in-person sex work” is giving way to new dynamics in which the “liquid body” circulates freely through videos, photos, and live interactions [61,62]. However, this does not mean that in-person sex, including paid encounters, has ceased to exist; on the contrary, digital platforms often function as amplified showcases that increase the visibility of AEWs and may culminate in in-person encounters, whether driven by audience demand or by commercial strategies inherent to the contemporary sex market [14].

The body of the adult entertainment worker thus becomes simultaneously a commodity, an artistic expression, a means of livelihood, and, in many cases, a platform for social influence [63].

This new role of digital influencers attributed to many AEWs represents a unique opportunity for public policy. These individuals have significant reach on social media, the ability to engage diverse audiences, and credibility within the communities they represent [64,65]. Integrating them as agents of community education could be an innovative and effective strategy to expand knowledge about PrEP, reduce the stigma associated with its use, and promote safer prevention practices while respecting the specific realities and languages of this sphere. To this end, it is recommended that public authorities establish strategic partnerships with influencers in this segment, developing targeted, culturally appropriate messages disseminated across multiple digital platforms.

Finally, any formulated policy must be grounded in the principles of intersectionality, recognizing that gender, sexual orientation, gender identity, race/ethnicity, socioeconomic status, and involvement in the digital market overlap, shaping the specific vulnerabilities of AEWs. Only approaches that take this complexity into account can ensure the effectiveness of PrEP as a tool for prevention and harm reduction, contributing to achieving global targets for eliminating HIV as a public health problem.

## 5. Limitations

Despite its relevance, this study has some limitations. Its cross-sectional design does not allow for causal inference, and the use of self-reported data may be subject to recall and social desirability biases. Furthermore, the use of convenience and snowball sampling introduces selection bias and limits the generalizability of the findings to the broader population of AEWs in Brazil. This sampling method may have particularly affected the representation of non-binary gender identities and individuals with limited access to healthcare services and information about PrEP, such as trans people who often face social marginalization, institutional stigma, and digital exclusion.

Additionally, many participants reported combining sex work with other forms of employment, which may influence the interpretation of results related to economic vulnerability and exposure to HIV risk. These factors highlight the need for future research employing probabilistic sampling and broader territorial coverage to better capture the diversity of this population and reduce potential under- or overestimations in subgroup analyses.

Despite these limitations, the study provides concrete insights to inform the development of more inclusive and effective public policies. Expanding access, reducing institutional stigma, and providing ongoing training for healthcare teams are essential strategies for improving engagement with HIV prevention services and ensuring better health outcomes for AEWs in Brazil.

## 6. Conclusions

Our study demonstrates that both cisgender AEWs and transgender individuals experience multiple, intersecting vulnerabilities that directly influence PrEP use. Our findings contribute to a still limited body of literature and underscore the need for intersectional approaches that consider not only issues of gender identity but also broader social determinants. It is important to emphasize that we are working with a concept—adult entertainment workers—that is in constant transformation, especially in the context of the rise of digital platforms, which have revolutionized the way sexual content is produced, marketed, and consumed. The emergence of “liquid bodies,” collaborations among content creators, and the growing normalization of practices such as condomless sex in pornography reflect significant changes in sexual economies and risk landscapes. These new dynamics highlight that PrEP interventions cannot remain anchored solely in traditional models of sex work or healthcare service delivery.

Although the availability of PrEP is a fundamental achievement within the framework of the SUS in Brazil, structural and contextual factors profoundly shape its adherence and sustained use. Overcoming these barriers requires inclusive and equitable public policies aligned with the contemporary realities of work in adult entertainment, whether in-person or digital. Engaging AEWs as digital influencers and leveraging their unique reach for health communication strategies and community education about PrEP represents an innovative and promising approach.

Furthermore, it is essential that healthcare services are prepared to welcome this population with respect, non-judgmental care, and continuous support, establishing trust that facilitates both the initiation and sustained use of PrEP. Personalized and integrated strategies for combination prevention should be prioritized, including addressing emerging phenomena such as chemsex, especially in contexts marked by multiple social vulnerabilities. Addressing these complexities is essential to ensure that PrEP fulfills its potential as a powerful tool in HIV prevention.

## Figures and Tables

**Table 1:** Bivariate analysis of sociodemographic factors associated with PrEP use among adult entertainment workers, Brazil, 2024.

Variable		PrEP Use				Total (n = 254)		PR (95%CI)	*p*-Value
		Yes (n = 83)		No (n = 171)					
		n	%	n	%	n	%		
Young adult (18 to 30 years)	Yes	44	31.2	97	68.8	141	55.5	0.90 (0.63–1.29)	0.576
	No [ref]	39	34.5	74	65.5	113	44.5		
Dependent on public health services	Yes	83	33.5	165	66.5	248	97.6	-	0.306 *
	No [ref]	-	-	4	100.0	4	1.6		
	Missing	-	-	-	-	2	0.8		
Has a steady partner	Yes	11	26.8	30	73.2	41	16.1	0.79 (0.46–1.36)	0.383
	No [ref]	72	33.8	141	66.2	213	83.9		
White race/ethnicity	Yes	44	40.0	66	60.0	110	43.3	1.49 (1.05–2.13)	0.026
	No [ref]	38	26.8	104	73.2	142	55.9		
	Missing	-	-	-	-	2	0.8		
Cisgender identity	Yes	57	38.5	91	38.5	148	58.3	1.54 (1.04–2.28)	0.025
	No [ref]	26	25.0	78	75.0	104	40.9		
	Missing		-	-	-	2	0.8		
Heterosexual	Yes [ref]	41	27.0	111	73.0	152	59.8	1.53 (1.08–2.17)	0.018
	No	42	41.2	60	58.8	102	40.2		
Has a disability	Yes	1	50.0	1	50.0	2	0.8	1.54 (0.38–6.22)	0.660
	No [ref]	81	32.5	168	67.5	249	98.0		
	Missing		-	-	-	3	1.2		
Less than 12 years of education	Yes [ref]	46	28.4	116	71.6	162	63.8	1.42 (0.99–2.01)	0.054
	No	37	40.2	55	59.8	92	36.2		
Home ownership	Yes [ref]	18	18.8	78	81.3	96	37.8	2.23 (1.41–3.52)	<0.001
	No	64	41.8	89	58.2	153	60.2		
	Missing		-	-	-	5	2.0		
Receives government assistance	Yes	11	39.3	17	60.7	28	11.0	1.23 (0.75–2.02)	0.439
	No [ref]	72	32.0	153	68.0	225	88.6		
	Missing		-	-	-	1	0.4		
Monthly income below BRL 2000	Yes	32	30.5	73	69.5	105	41.3	0.80 (0.55–1.17)	0.250
	No [ref]	41	38.0	67	62.0	108	42.5		
	Missing		-	-	-	41	16.1		
Adult entertainment is main occupation	Yes	54	69.2	24	30.8	78	30.7	4.01 (2.79–5.76)	<0.001
	No [ref]	29	17.3	139	82.7	168	66.1		
	Missing		-	-	-	8	3.1		
Changed occupation during the COVID-19 pandemic	Yes	52	40.0	78	60.0	130	51.2	1.42 (0.97–2.09)	0.064
	No [ref]	27	28.1	69	71.9	96	37.8		
	Missing		-	-	-	28	11.0		

* ref: reference exposure category. 95%CI: 95% confidence interval. PR: crude prevalence ratio. Fisher’s Exact Test. Source: Prepared by the authors, 2024.

**Table 2:** Bivariate analysis of PrEP use among adult entertainment workers according to sexual practices and lifestyle habits, Brazil, 2024.

Variables		PrEP Use				Total (n = 254)		PR (95%CI)	*p*-Value
		Yes (n = 83)		No (n = 171)					
		n	%	n	%	n	%		
Practiced social isolation during the COVID-19 pandemic	Yes	52	30.6	118	69.4	170	66.9	0.82 (0.56–1.20)	0.319
	No [ref]	25	37.3	42	62.7	67	26.4		
	Missing	-	-	-	-	17	6.7		
Received ≥3 doses of COVID-19 vaccine	Yes	61	35.3	112	64.7	173	68.1	1.17 (0.78–1.75)	0.438
	No [ref]	22	30.1	51	69.9	73	28.7		
	Missing	-	-	-	-	8	3.1		
Alcohol consumption	Yes	59	33.5	117	66.5	176	69.3	1.09 (0.73–1.63)	0.659
	No [ref]	23	30.7	52	69.3	75	29.5		
	Missing	-	-	-	-	3	1.2		
Illicit drug use	Yes	41	39.0	64	61.0	105	41.3	1.60 (0.69–3.69)	0.073
	No [ref]	41	28.3	104	71.7	145	57.1		
	Missing	-	-	-	-	4	1.6		
History of violence	Yes	43	34.7	81	65.3	124	48.8	1.39 (0.90–2.13)	0.127
	No	23	25.0	69	75.0	92	36.2		
	Missing	-	-	-	-	38	15.0		
Engages in receptive anal intercourse	Yes	2	25.0	6	75.0	8	3.1	0.76 (0.23–2.58)	1.000
	No [ref]	69	32.7	142	67.3	211	83.1		
	Missing	-	-	-	-	35	13.8		
History of STI	Yes	50	56.2	39	43.8	89	35.0	2.83 (1.97–4.05)	<0.001
	No	32	19.9	129	80.1	161	63.4		
	Missing	-	-	-	-	4	1.6		
Use of other combined prevention strategies	Yes	78	45.1	95	54.9	173	68.1	7.67 (2.92–20.11)	<0.001 *
	No [ref]	4	5.9	64	94.1	68	26.8		
	Missing	-	-	-	-	13	5.1		
History of PEP use	Yes	55	69.6	24	30.4	79	31.1	4.55 (3.11–6.67)	<0.001
	No [ref]	26	15.3	144	84.7	170	66.9		
	Missing					5	2.0		
Up-to-date immunizations	Yes	65	34.2	125	65.8	190	74.8	1.15 (0.74–1.79)	0.537
	No [ref]	17	29.8	40	70.2	57	22.4		
	Missing	-	-	-	-	7	2.8		
Currently under medical treatment	Yes	52	39.7	79	60.3	131	51.6	1.56 (1.07–2.27)	0.017
	No [ref]	30	25.4	88	74.6	118	46.5		
	Missing	-	-	-	-	5	2.0		
Visited a doctor in the past 6 months	Yes	69	36.7	119	63.3	188	74.0	1.71 (0.93–3.15)	0.071 *
	No [ref]	9	21.4	33	78.6	42	16.5		
	Missing	-	-	-	-	24	9.4		
Visited a dentist in the past 6 months	Yes	38	44.2	48	55.8	86	33.9	1.67 (1.15–2.41)	0.007
	No [ref]	35	26.5	97	73.5	132	52.0		
	Missing	-	-	-	-	36	14.2		
Experienced violence or discrimination in healthcare	Yes	18	34.6	34	65.4	52	20.5	1.04 (0.68–1.59)	0.844
	No [ref]	65	33.2	131	66.8	196	77.2		
	Missing	-	-	-	-	6	2.4		

* [ref] reference exposure category. 95%CI: 95% confidence interval. PR: crude prevalence ratio. Fisher’s Exact Test. Source: Prepared by the authors, 2024.

**Table 3:** Multivariate analysis of factors independently associated with PrEP use among adult entertainment workers, Brazil, 2024.

Variables	Β	aPR	95%CI		*p*-Value
			Lower	Upper	
Intercept	−2.396	0.09	0.06	0.14	<0.001
Main occupation in adult entertainment	0.991	2.69	1.84	3.95	<0.001
History of PEP use	0.914	2.49	1.63	3.81	<0.001
Currently under medical treatment	0.445	1.56	1.15	2.12	0.005
History of STI	0.416	1.51	1.10	2.08	0.010

Adjusted for age. Omnibus test (*p* < 0.001). Deviance: 112.63. Akaike Information Criterion (AIC = 298.63). 95%CI: 95% confidence interval. aPR: adjusted prevalence ratio. Source: Prepared by the authors, 2024.

## Data Availability

Data connected to this research are available from the corresponding author upon request (Á.F.L.d.S.).
